# Role of Calcium Signals on Hydrogen Peroxide-Induced Apoptosis in Human Myeloid HL-60 Cells

**Published:** 2009-09

**Authors:** Ignacio Bejarano, Javier Espino, David González-Flores, Javier G. Casado, Pedro C. Redondo, Juan A. Rosado, Carmen Barriga, José A. Pariente, Ana B. Rodríguez

**Affiliations:** *Department of Physiology, University of Extremadura, Badajoz, Spain*

**Keywords:** caspases, calcium signal, apoptosis, mitochondria, H_2_O_2_, HL-60 cells

## Abstract

The present study is aimed to determine the role of calcium signaling evoked by the oxygen radical, hydrogen peroxide (H_2_O_2_) and the specific inhibitor of calcium reuptake thapsigargin on apoptosis in the human leukemia cell line HL-60. Our results show that treatment of HL-60 cells with 100 µM H_2_O_2_ and 1 µM thapsigargin induced a transient increase in cytosolic free calcium concentration ([Ca^2+^]_c_) due to calcium release from internal stores. These stimulatory effects on calcium signals were followed by activation of the mitochondrial permeability transition pore (mPTP), as well as a time-dependent increase in caspase-9 and -3 activities. Our results also show that H_2_O_2_ and thapsigargin were able to increase the relative content of fragmented DNA and phosphatidylserine externalization, as detected by double-staining with propidium iodide (PI) and annexin-V-FITC, respectively. Treatment of cells with H_2_O_2_ or thapsigargin resulted in activation of the proapoptotic protein Bid. Furthermore, coimmunoprecipitation experiments showed active Bax was bound to Bid, which regulates Bid activity and promotes apoptosis. Our findings suggest that H_2_O_2^−^_ and thapsigargin-induced apoptosis is dependent on rises in [Ca^2+^]_c_ in human myeloid HL-60 cells.

## INTRODUCTION

Apoptosis is a gene-regulated form of cell death that is critical for normal development and tissue homeostasis. A major component of the apoptotic machinery involves a family of aspartic acid-directed cysteine proteases, called caspases (cysteinyl aspartate-specific proteinases), which cleave multiple protein substrates *en masse*, leading to the loss of cellular structure and function and ultimately resulting in cell death (1).

From a functional point of view, caspases are involved in apoptosis acting both initiators (caspase 8, 9, and 10) and effectors (caspase 3, 6, and 7) (2). Caspase 8 was identified as the most important initiator enzyme of the Fas/CD95 pathway (3). Caspase 9 interacts with many other regulators and transducers, such as cytochrome c, in intrinsic pathway (4). Both initiator caspases are activators of downstream caspases. Caspase 3, the most important among them, executes the final disassembly of the cell by cleaving a variety of cell structure proteins and generating DNA strand breaks (5).

Traditionally, two apoptotic pathways have been described. One pathway is the so-called extrinsic pathway initiated by the binding of an extracellular death ligand, such as FasL, to its cell-surface death receptor, such as Fas (6). The second pathway is the intrinsic pathway, which is mediated by mitochondrial alterations. In response to apoptotic stimuli, several proteins are released from the intermembrane space of mitochondria into the cytoplasm (7). Some of these well-characterized proteins include cytochrome c, which mediates the activation of caspase-9 (8), triggering in turn a cascade of caspase activation, including caspase-3, which promotes cellular self-destruction.

On the other hand, whereas ion calcium is a key regulator of cell survival, the sustained elevation of cytosolic free calcium concentration ([Ca^2+^]_c_) plays a role in cell death (9). The proapoptotic effects of calcium are mediated by a diverse range of calcium-sensitive factors that are compartmentalized in various intracellular organelles including endoplasmic reticulum and mitochondria (10). Excessive calcium load to the mitochondria may induce apoptosis by stimulating the release of apoptosis promoting factors from the mitochondrial intermembrane space to the cytoplasm and by impairing mitochondrial function (11).

Calcium-dependent increase in mitochondrial permeability to ions and solutes with molecular masses up to 1500 Da, matrix swelling, and uncoupling of oxidative phosphorylation, which has been defined as permeability transition (12), is currently ascribed to the opening of the mitochondrial permeability transition pore (mPTP) (13). The mPTP is a megachannel formed in the mitochondrial membranes which is found at the contact sites between inner and outer mitochondrial membranes (14, 15). Under conditions like oxidative stress, calcium overload, and low ATP levels, a number of members of Bcl-2 family are recruited to enable the pore formation at its high conductance state (14, 15).

Furthermore, mitochondria are the major source of reactive oxygen species (ROS) in the cell. Superoxide (O_2_^−•^) is generated by the operation of complexes I and III in the matrix and is converted to hydrogen peroxide (H_2_O_2_) by Mn-superoxide dismutase. Apoptosis can also be stimulated by ROS in several cell types (16–18). Recently, it has been reported that ROS can mediate the apoptosis induced by either growth factors, such as transforming growth factor-β (TGF-β) in human lens epithelial cells (19), or classical agonists, such as thrombin in human platelets (20) or progesterone in human spermatozoa (21).

Although ROS and calcium have been separately reported to be important mediators of apoptosis, little correlation between these two mediators has been reported. Here, we focused on the role of intracellular calcium in H_2_O_2_-induced apoptosis in human leukemia cell line HL-60 in culture, analyzing caspase-3 and -9 activation, mitochondrial depolarization, induction of the mitochondrial permeability transition pore and activation of the proapoptotic proteins Bax and Bid.

## MATERIALS AND METHODS

### Materials

HL-60 15-12 cell line (ECACC N° 88120805), a variant of HL-60 which is differentiating towards either neutrophils or monocytes was purchased from The European Collection of Cell Cultures (ECACC) (Dorset, U.K.). Fetal bovine serum (FBS) and penicillin/streptomycin were obtained from HyClone (Aalst, Belgium). L-Glutamine, RPMI 1640 medium were obtained from Cambrex (Verviers, Belgium). Dimethyl sulfoxide (DMSO), thapsigargin, N-acetyl-Asp-Glu-Val-Asp-7-amido-4-methylcoumarin (AC-DEVD-AMC), dithiothreitol (DTT) and anti-Bax monoclonal antibody (Clone 6A7) were obtained from Sigma (Madrid, Spain). N-acetyl-Leu-Glu-His-Asp-7-amido-4-trifluoromethylcoumarin (AC-LEHD-AMC) was from Bachem (Bubendorf, Switezerland). Calcein-AM and fura-2 acetoxymethyl ester (fura-2/AM) were obtained from Molecular Probes (Barcelona, Spain). Propidium iodide (PI) and annexin-V-FITC were from Immunostep (Salamanca, Spain). Horseradish peroxidase-conjugated goat anti-rabbit IgG antibody was from Santa Cruz (Santa Cruz, CA, U.S.A.). Protein A-agarose and horseradish peroxidase-conjugated sheep anti-mouse IgG antibody were from Upstate Biotechnology Inc. (Madrid, Spain). Anti-Bid antibody was from Cell Signaling (Barcelona, Spain). Hyperfilm ECL was from Amersham (Arlington Heights, IL, U.S.A.). All other reagents were of analytical grade.

### Cellular culture

Human promyelocytic leukemia HL-60 cells were grown in RPMI 1640 medium supplemented with 2 mM L-glutamine, 10% heat-inactivated fetal bovine serum, 100 U/mL penicillin, 100 µg/mL streptomycin and 1.25% DMSO at 37°C under a humidified condition of 95% air and 5% CO_2_. Cells were routinely plated at a density of 3 × 10^5^ cells/ml into fresh flasks and the viability was >95% in all experiments as assayed by the trypan-blue exclusion method.

### Measurement of cytosolic free calcium concentration ([Ca^2+^]_c_)

Cells were loaded with fura-2 by incubation with 2 μM fura-2 acetoxymethyl ester (fura-2/AM) for 45 minutes at 37°C according to a procedure published elsewhere (22). Once loaded, cells were washed and gently resuspended in Na-HEPES solution containing (in mM): NaCl, 140; KCl, 4.7; CaCl_2_, 1.2; MgCl_2_, 1.1; glucose, 10; and HEPES, 10 (pH 7.4). Fluorescence was recorded from 2 mL aliquots of magnetically stirred cellular suspension (2 × 10^6^ cells/mL) at 37ºC by using a spectrofluorophotometer (Shimadzu RF-5301-PC) with excitation wavelengths of 340 and 380 nm and emission at 505 nm. Changes in [Ca^2+^]_c_ were monitored by using the fura-2 340/380 nm fluorescence ratio and were calibrated according to the method of Grynkiewicz *et al*. (23). In the experiments where calcium-free medium is indicated, calcium was omitted and 1 mM ethylene glycol-bis(2-aminoethylether)-N,N,N′,N′-tetraacetic acid (EGTA) was added.

### Assay for caspase activity

To determine caspase-3 and -9 activity, stimulated or resting cells were sonicated and cell lysates were incubated with 2 mL of substrate solution (20 mM HEPES, pH 7.4, 2 mM EDTA, 0.1% CHAPS, 5 mM DTT and 8.25 µM of caspase substrate) for 1 h at 37 °C as previously described (24). The activities of caspase-3 and -9 were calculated from the cleavage of the respective specific fluorogenic substrate (AC-DEVD-AMC for caspase-3 and AC-LEHD-AMC for caspase-9). Substrate cleavage was measured with a fluorescence spectrophotometer with excitation wavelength of 360 nm and emission at 460 nm. Preliminary experiments reported that caspase-3 or -9 substrate cleaving was not detected in the presence of the inhibitors of caspase-3 or -9, DEVD-CMK or z-LEHD-FMK, respectively. The data were calculated as fluorescence units/mg protein and presented as fold increase over the pretreatment level (experimental/control).

### Calcein and Cl_2_Co co-loading

Cells were loaded with 1 µM calcein-AM in the presence of 1 mM Cl_2_Co during 15 minutes at 37°C and later were washed using always the same RPMI medium where the cells had been grown. Mitochondrial calcein fluorescence was measured with a fluorescence spectrophotometer with excitation wavelength of 488 nm and emission at 515 nm (25). Data are expressed as fractional changes of emitted fluorescence (F/F_0_).

### Annexin-V staining

Cells were harvested through and washed twice with PBS and centrifuged at 500 g for 5 min, then the supernatant was discarded and the pellet was resuspended in 95 μL annexin-V-binding buffer at a density of 10^5^–10^6^ cells/mL with 2.5 µg/mL annexin-V-FITC at 4°C for 30 min. Cells were analyzed by flow cytometry on a FACScan cytometer (BD Biosciences) after addition of 2.5 µg/mL propidium iodide (PI). 10,000 events were analyzed using the FL-1 and FL-3 detector filters. Each sample was tested 3 to 5 times in independent experiments. Annexin-V binds to those cells that express phosphatidylserine on the outer layer of the cell membrane, and PI stains the cellular DNA of those cells with a compromised cell membrane. This allows for live cells (unstained with either fluorochrome) to be discriminated from apoptotic cells (stained only with annexin-V) and necrotic cells (stained with both annexin-V and PI).

### Cell cycle analysis

After treatment, cells (approximately 1 × 10^6^ cells/mL) were washed with PBS and fixed in 70% ethanol for 30 min at 4°C. The cells were again rinsed with PBS and resuspended in 500 µL PBS containing 2.5 µg/mL PI and 50 µg/mL RNase. The samples were kept in the dark at 4°C for 30 min and analyzed by flow cytometry on a FACScan cytometer (BD Biosciences) using the FL-2 detector filter. Cells undergoing apoptosis stain with PI and exhibit a reduced DNA content with a peak in the hypodiploid region (26) The percentages of every phase (G2/M, S, and G0/G1) were analyzed and the percentage of apoptosis was taken as the fraction with hypodiploid DNA content.

### Immunoprecipitation

Bax activation and association with Bid were determined by immunoprecipitation as previously described (27). Briefly, HL-60 cell suspensions were stimulated, as indicated, and lyzed by mixing equal volume of cell sample with a radio immunoprecipitation assay buffer (RIPA 2x). Following preparation of cell lysates, protein concentration was determined using the Bradford assay and adjusted to a protein concentration of 50 µg/mL with lysis buffer. Bax was immunoprecipitated from cell lysates by incubation with 2 μg of anti-Bax antibody (clone 6A7) and protein A-agarose overnight at 4°C on a rocking platform. Immunoprecipitates were resolved by 15% SDS-PAGE and Western blotting was performed as described in the following section.

### Western blotting

Bax and Bid expression in HL-60 cells was analyzed by Western Blotting. Briefly, HL-60 cell suspensions were stimulated, as indicated, and lyzed by mixing with the appropriate amount of Laemmli buffer. Following preparation of cell lysates, protein concentration was determined using the Bradford assay and adjusted to a protein concentration of 50 µg/mL with lysis buffer. Proteins were separated by 15% SDS-PAGE and electrophoretically transferred, for 2 h at 0.8 mA/cm^2^, in a semi-dry blotter (Hoefer Scientific, Newcastle, Staffs., U.K.) onto nitrocellulose for subsequent Western blotting. Non-specific protein binding sites of the nitrocellulose membranes were blocked by incubating overnight with 10% (w/v) BSA in Tris-buffered saline with 0.1% Tween 20 (TBST). Membranes were incubated with the anti-Bax antibody diluted 1:200 in TBST for 2 h or with the anti-Bid antibody diluted 1:500 overnight. To detect the primary antibodies, blots were incubated with horseradish peroxidase-conjugated sheep anti-mouse IgG antibody diluted 1:5000 or horseradish peroxidase-conjugated donkey anti-rabbit IgG antibody diluted 1:10000 in TBST, respectively, and exposed to enhanced chemiluminescence reagents for 5 min. Blots were then exposed to photographic films and the optical density was estimated using scanning densitometry.

### Statistical Analysis

Data are presented as mean ± standard error of mean (S.E.M.) and analysis of statistical significance was performed using Student's t-test. *p*<0.05 was considered to indicate a statistically significant difference.

## RESULTS

### Effects of H_2_O_2_ on calcium mobilization

**Figure 1:**
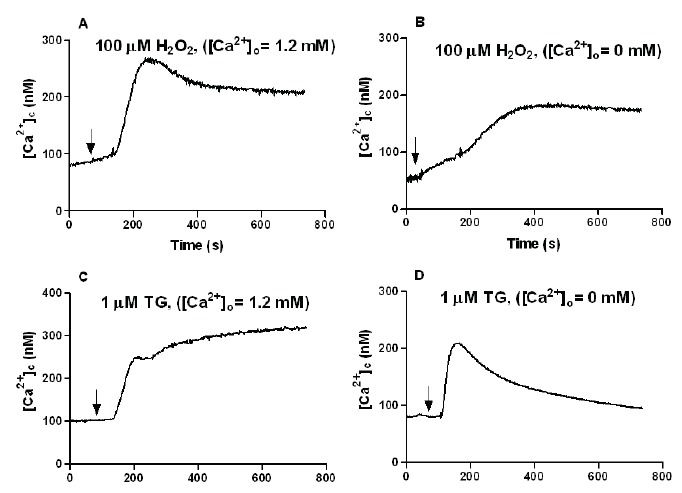
Effect of H_2_O_2_ on [Ca^2+^]_c_. Fura-2-loaded HL-60 cells were stimulated with 100 µM H_2_O_2_ (A and B) or 1 µM thapsigargin (TG) (C and D) as indicated by arrows in a calcium-normal ([Ca^2+^]_0_=1.2 mM) (A and C) or -free solution ([Ca^2+^]_0_=0 mM + 1 mM EGTA) (B and D). Traces are representative of 5–7 independent experiments.

In the presence of normal extracellular calcium concentration, fura-2-loaded HL-60 cells were treated with the oxygen radical H_2_O_2_. As shown in Figure 1A, stimulation with 100 µM H_2_O_2_ caused a slow and sustained [Ca^2+^]_c_ increase, which reached a stable [Ca^2+^]_c_ plateau after 5–7 minutes of administration. Figure 1 also demonstrates that the increase of [Ca^2+^]_c_ induced by 100 µM H_2_O_2_ was also observed in calcium-free medium (Figure 1B), reflecting the release of calcium from intracellular store(s), though the calcium signal was smaller compared to that obtained in the presence of extracellular calcium. As expected, stimulation of HL-60 cells with the specific inhibitor of calcium reuptake thapsigargin (1 µM) in the presence of normal extracellular calcium concentration, caused a typical transient increase in [Ca^2+^]_c_, which reached a stable [Ca^2+^]_c_ plateau after 4–5 minutes of stimulation (Figure 1C). This increase induced by thapsigargin was also observed in a calcium-free medium (Figure 1D), also reflecting the release of calcium from internal pools.

### H_2_O_2_ induces activation of caspase-3 and -9

**Figure 2:**
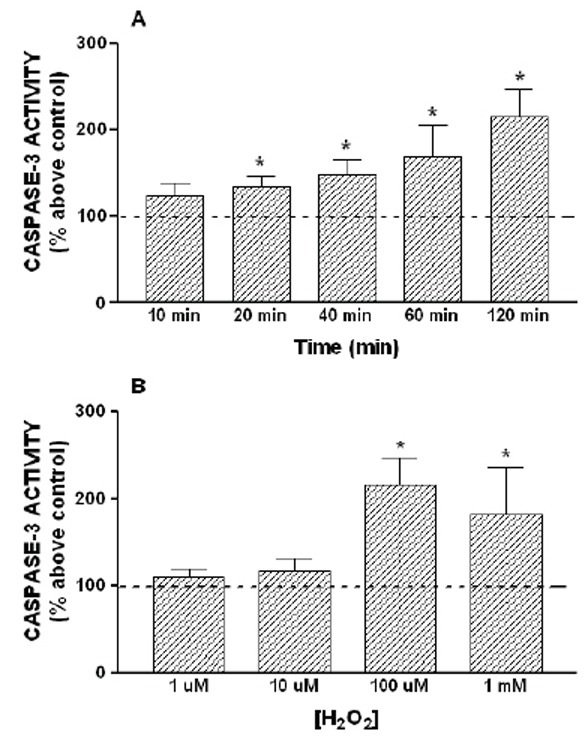
Time course and concentration dependence of H_2_O_2_-induced activation of caspase-3. HL-60 cells were stimulated for various periods of time (10–120 minutes) with 100 μM H_2_O_2_ (A) or for 120 minutes with increasing concentration of H_2_O_2_ (1 µM–1mM) (B). Caspase-3 activity was estimated as described under “Material and Methods”. Values are presented as means ± SEM of 5–8 separate experiments and expressed as percentage above control (untreated samples). *p<0.05 compared to control values.

**Figure 3:**
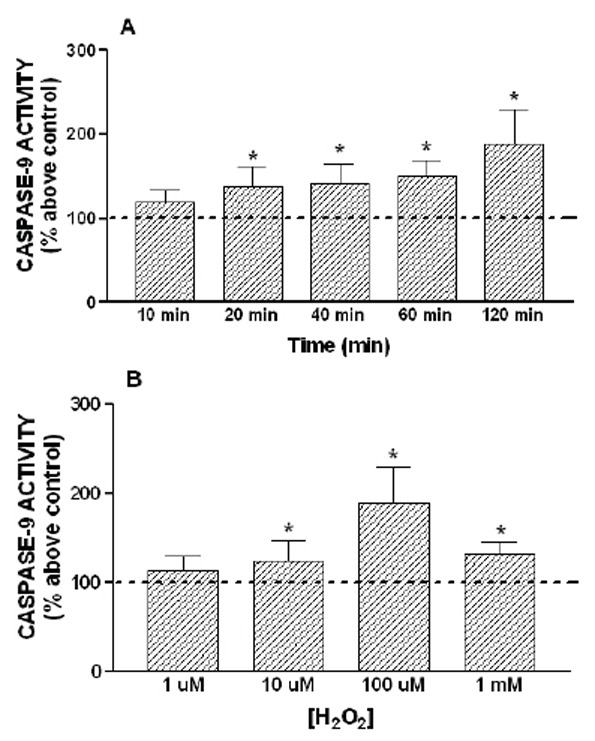
Time course and concentration dependence of H_2_O_2_-induced activation of caspase-9. HL-60 cells were stimulated for various periods of time (10–120 minutes) with 100 μM H_2_O_2_ (A) or for 120 minutes with increasing concentration of H_2_O_2_ (1 µM–1 mM) (B). Caspase-9 activity was estimated as described under “Material and Methods”. Values are presented as means ± SEM of 4–6 separate experiments and expressed as percentage above control (untreated samples). *p<0.05 compared to control values.

To examine the effect of H_2_O_2_ on caspase-3 activation, HL-60 cells were treated with 100 µM H_2_O_2_ for 10–120 min. As shown in Figure 2A, treatment of cells with 100 µM H_2_O_2_ induced a time-dependent activation of caspase-3. Cell stimulation with 100 µM H_2_O_2_ caused a detectable activation of caspase-3 after 20 min of treatment with a 134 ± 12 % above control, and the maximum effect was obtained after 120 min of stimulation (215 ± 31 % above control, Figure 2A, p<0.05). Caspase-9 is an initiator caspase that is involved in the initial steps of mitochondrial apoptosis (9). To investigate whether the activation of caspase-3 induced by H_2_O_2_ is a mitochondrial apoptosis, we checked the caspase-9 activity in the presence of 100 µM H_2_O_2_. Consistent with the results presented above, activation of caspase-9 by 100 µM H_2_O_2_ showed a similar activation pattern to that of caspase-3, reaching a maximum peak of caspase-9 activity after 120 min of stimulation, with a 189 ± 40 % above control (Figure 3A).

**Figure 4:**
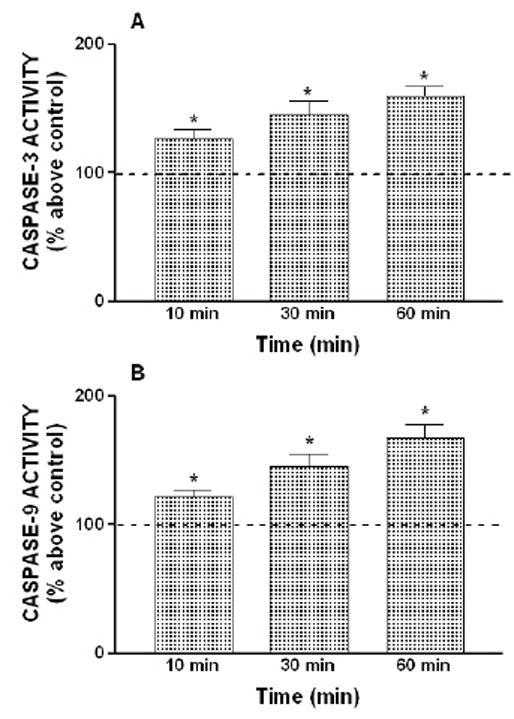
Time course dependence of thapsigargin-induced activation of caspase-3 and -9. HL-60 cells were stimulated for various periods of time (10–60 minutes) with 1 μM thapsigargin (TG). Caspase-3 (A) and -9 (B) activities were estimated as described under “Material and Methods”. Values are presented as means ± SEM of 5 separate experiments and expressed as percentage above control (untreated samples). *p<0.05 compared to control values.

However, the effect of H_2_O_2_ on caspase-3 and -9 activation was not clearly concentration-dependent, as shown in Figures 2B and 3B. In fact, treatment for 120 min at 1 µM or 10 µM was unable to induce activation of caspase-3 and -9. The maximun effect was obtained with 100 µM of H_2_O_2_, while 1 mM H_2_O_2_ induced a lower activation of the caspase-3 and -9 than 100 µM H_2_O_2_ (Figure 2B and 3B). In addition, the treatment of HL-60 cells with 1 µM thapsigargin, a sarco-endoplasmic reticulum calcium ATPase (SERCA) inhibitor, which depletes the intracellular calcium stores, was also able to induce a time-dependent activation of caspase-3 and -9, reaching a maximal caspases activities after 60 minutes of stimulation (Figure 4).

### Activation of mPTP by H_2_O_2_

**Figure 5:**
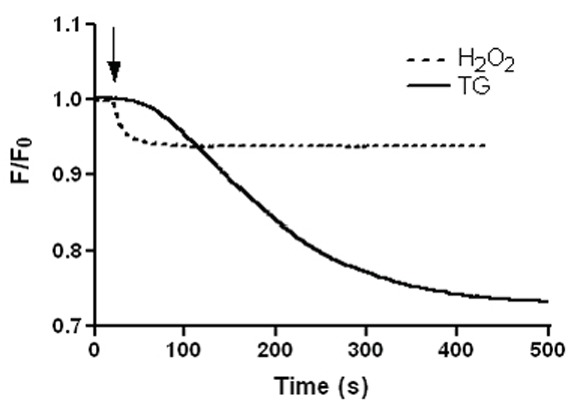
H_2_O_2_ causes a loss of mitochondrial staining by calcein. Calcein-loaded cells in the presence of 1 mM Cl_2_Co were stimulated with 100 μM H_2_O_2_ or 1 µM thapsigargin (TG). Changes in calcein fluorescence were detemined as shown under “Material and Methods” and are expressed as fractional changes of emitted fluorescence (F/F_0_). Traces are representative of 5–6 independent experiments.

To further investigate the role of H_2_O_2_ in mitochondrial apoptosis, we probed mPTP opening in HL-60 cells loaded with calcein-AM in the presence of cobalt chloride to quench fluorescence from all cellular domains except from within mitochondria (25, 30). Using this protocol, 100 µM H_2_O_2_ caused a loss of mitochondrial calcein fluorescence (Figure 5). Similar results, although to a larger extent, were obtained when the cells were treated with 1 µM thapsigargin (Figure 5), suggesting that H_2_O_2_ and thapsigargin cause permeability transition pore induction in mitochondria. Although this protocol does not distinguish between calcein efflux and cobalt influx, it is consistent with induction of the permeability transition pore.

### Effects of H_2_O_2_ on cell cycle

**Figure 6:**
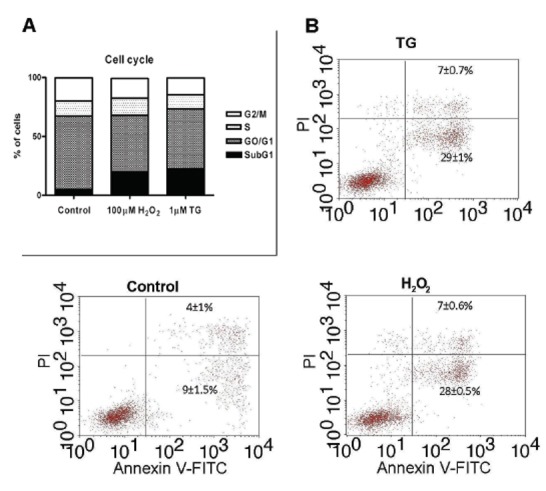
Effect of H_2_O_2_ on apoptosis induction in HL-60 cells. Cells were stimulated with 100 µM H_2_O_2_ for 120 min or 1 µM thapsigargin (TG) for 60 min. The cell cycle phase distribution was determined by flow cytometry analysis using ethanol-fixed, propidium iodide (PI)-stained cells (A). To determine the relationship between apoptosis and necrosis, cells were stained with annexin-V-FITC and propidium iodide (PI) and analyzed by flow cytometry (B) as described under “Material and Methods”. Values are presented as mean percentages ± S.E.M. of 3–4 separate experiments.

To evaluate the effect of H_2_O_2_ on cell cycle, cells were stained with PI which has affinity for nucleic acids, and incubated with RNase in presence of triton X-100, 0.01% as nonionic detergent which supplies the PI staining. An increase in hypodiploid DNA content was found in H_2_O_2_-treated cells. Treatment with 100 µM H_2_O_2_ for 120 minutes showed an important Sub-G1 arrest at expense of the rest of the phases, mainly G0/G1 was decreased after the H_2_O_2_ treatment compared with the control group (Figure 6A). On the other hand, treatment of cells with 1 µM thapsigargin for 60 minutes shows similar results, which consisted in an increase of sub-G1 and a decrease in other phases compared with the control group (Figure 6A).

Annexin-V-FITC and PI stainings were used to detect apoptotic and necrotic changes in H_2_O_2_-treated HL-60 cells. Double-stained cells were analyzed by flow cytometry and the results revealed that a 9 ± 1.5 % of control cells had only annexin-V positivity (early apoptotic cells) and 4 ± 1 % annexin-V/PI positivity (necrotic cells) (Figure 6B). As also shown in Figure 6B, 100 µM H_2_O_2_ induced an increase in the percentage of apoptosis. The population of early apoptotic cells increased after 120 minutes treatment with 100 µM H_2_O_2_ (28 ± 0.5%, Figure 6B). In addition, the treatment of cells with 100 µM H_2_O_2_ for 120 minutes induced a slight increase in the population of necrotic cells as compared with the control group (7 ± 0.6 % versus 4 ± 1%). For comparison, the effect of administration of 1 μM thapsigargin for 60 minutes is included in Figure 6. Thapsigargin treatment increased in the same way the number of apoptotic cells in a 29 ± 1%, as revealed by annexin-V assay, and the percentage of necrotic cells was 7 ± 0.7% (Figure 6B). These results indicate that the treatments with H_2_O_2_ and thapsigargin trigger cells to apoptosis.

### Effects of H_2_O_2_ on activation of Bax and Bid

**Figure 7:**
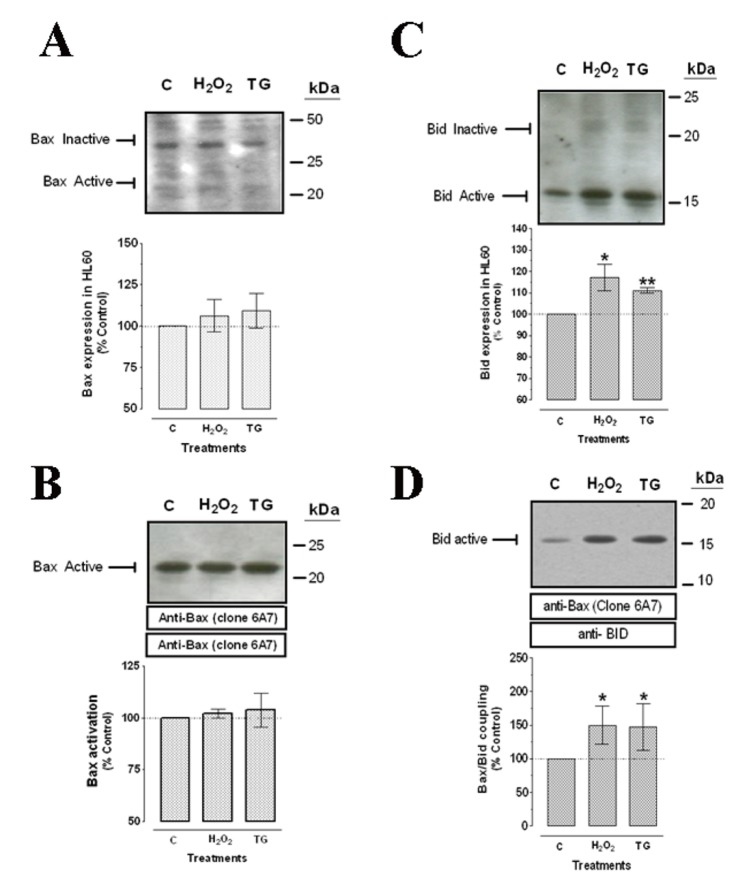
Effects of H_2_O_2_ and thapsigargin on Bax activation and association with Bid. HL-60 cells were treated with 100 µM H_2_O_2_ for 120 minutes or 1 µM thapsigargin (TG) for 60 minutes, and then lyzed. Samples were either analyzed by western blotting (A and C) or immunoprecipitated with anti-Bax antibody (clone 6A7; B and D). Protein expression, activity and association were analyzed by Western blotting using an anti-Bax antibody (A and B) or an anti-Bid antibody (C and D) as described under “Material and Methods”. Histograms show Bax and Bid expression (A and C) and Bax activation or Bax/Bid association (B and D), respectively. Data are expressed as percentage of control (untreated cells) and presented as mean ± S.E.M. of 4 independent experiments. *p<0.05 and **p<0.01 compared to control values.

We have further investigated whether the expression of the proapototic proteins, Bax and Bid may be altered by treatment of HL-60 cells with H_2_O_2_. Bid activation was analyzed by Western blotting using a rabbit anti-Bid antibody that detects both the cleaved (active) and native forms of Bid (31). Active Bax was detected by immunoprecipitation with the anti-Bax antibody (clone 6A7), which reacts only with Bax in its conformationally active state, followed by Western blotting with the same antibody as described previously (27, 32). Other forms of Bax were detected by Western blotting of the samples with the anti-Bax antibody (clone 6A7). As shown in Figure 7A, 100 µM H_2_O_2_ and 1 µM thapsigargin did not significantly modify the expression of Bax in these cells, detected as the sum of the amount of the forms of Bax detected at 42 and 21 kDa (after treatment with H_2_O_2_ or thapsigargin Bax expression increased by 106.3 ± 9.8% of control and 109.2 ± 10.6% of control, respectively). In contrast, Bid expression, estimated as described for Bax, was significant increased after treatment with both agents, with an increase of 117.0 ± 6.3% of control after treatment with 100 µM H_2_O_2_ and 111.1 ± 1.2% of control in cells treated with thapsigargin (*p*<0.05, Figure 7C). As shown in Figure 7B, incubation of HL-60 cells with either 100 µM H_2_O_2_ or 1 µM thapsigargin slightly enhanced Bax activity by 102.1 ± 2.1% of control and 103.6 ± 8.2% of control, respectively.

On the other hand, we also analyzed Bax activation and Bax/Bid association by co-immunoprecipitation using a mouse monoclonal anti-Bax antibody (clone 6A7) that recognises a specific region that is very close to the Bax dimerization domain that is only exposed once Bax is activated in its monomeric conformation (33). We found that HL-60 treatment with either 100 µM H_2_O_2_ for 120 minutes or 1 µM thapsigargin for 60 minutes, significantly increased Bid and Bax association in a 149.7 ± 28.6% of control and in a 147.1 ± 35.0% of control, respectively (Figure 7D, *p*<0.05).

## DISCUSSION

The proapoptotic effects of calcium are mediated by a diverse range of calcium-sensitive factors that are compartmentalized in various intracellular organelles including endoplasmic reticulum and mitochondria (28). It has been reported that a prolonged elevation in [Ca^2+^]_c_ and alterations in calcium homeostasis initiate the mitochondrial apoptotic pathway (9, 28) and induce endoplasmic reticulum stress that, in turn, leads to apoptosis (29). Excessive calcium load to the mitochondria may induce apoptosis by stimulating the release of apoptosis promoting factors from the mitochondrial intermembrane space to the cytoplasm and by impairing mitochondrial function (11, 34). Apoptosis can also be stimulated by ROS in several cell types (24–26); even it has been shown that H_2_O_2_ evokes increased [Ca^2+^]_c_ in the absence of extracellular calcium, indicating that H_2_O_2_ mobilizes calcium from intracellular stores (7, 21) leading cells into an apototic state.

This study was designed to determine the effects of the reactive oxygen species H_2_O_2_ and the specific inhibitor of calcium reuptake thapsigargin on mitochondrial apoptosis in the HL-60 tumor cell line, which is a useful cellular model to study cell growth of leukemia cells. Our results demonstrate that H_2_O_2_ and thapsigargin evoked apoptotic events through the increase of [Ca^2+^]_c_ in HL-60 cells, inducing caspase-9 and -3 activation, induction of the mPTP and activation of proapoptotic proteins.

Caspase-9 and -3 are major mediators of apoptotic cell death and their activation is widely considered an apoptotic marker. Here, we show that stimulation of HL-60 cells with H_2_O_2_ or thapsigargin increased caspase-3 activity in a time-dependent manner. These results are in agreement with previous studies, where the activation of executioner caspase-3 is reported by H_2_O_2_ and thapsigargin (35–37). According to Shi *et al*. (4) caspase-3 can be also activated by either extrinsic and by mitochondrial-mediated (intrinsic) pathway. Additionally, our results also demonstrate a time dependent activation of the initiator caspase-9 induced by administration of H_2_O_2_ or thapsigargin, which is indicating the mitochondrial involvement in the activation of caspase-3. In addition, our findings demonstrate that caspase activation evoked by H_2_O_2_ or thapsigargin is accompanied by induction of mPTP, which strongly suggests the mitochondrial participation of the intrinsic pathway of apoptosis. Our results are in agreement with studies which support that endogenous reactive oxygen species, such as H_2_O_2_, cause apoptosis through mPTP (38, 39), and an excessive intracellular [Ca^2+^] has been linked to activation of the mPTP and induction of apoptosis (40).

A number of studies showed that high concentration of H_2_O_2_ induces necrosis and low concentration induces apoptosis (41–43). Although apoptosis and necrosis have different effects on cellular physiology, the cellular response to H_2_O_2_ is continuum from apoptosis to necrosis (41). Our findings by flow cytometry indicate that the treatment of H_2_O_2_ for 120 minutes induces a significant increase in both the population of apoptotic cells and the population of necrotic cells, but the increase in the apoptotic population was notably higher than the necrotic one. Similar results were found when the intracellular calcium stores were depleted by thapsigargin, which showed a higher increase in the annexin-V stained population than double stained one (annexin-V and PI). This suggests that depletion of intracellular calcium stores by thapsigargin or oxidative stress resulting from H_2_O_2_ treatment induce early apoptotic state mainly in HL-60 cells. Alternatively, in this study we showed that the effects of H_2_O_2_ and thapsigargin on caspase activation are related with alteration in the cell cycle progression. In fact, depletion of intracellular calcium stores by H_2_O_2_ and thapsigargin reveals variations on cell cycle since the G2/M and S phases are arrested by the Sub-G1 one. This result indicates that H_2_O_2_ and thapsigargin have proapoptotic effects.

During apoptosis several members of the Bcl-2 family undergo activation. For instance, caspase 8 activation, via stimulation of Fas in the plasma membrane, induces rapid activation of Bid and evokes its translocation to mitochondria as tBid. tBid, with 15 kDa, is able to bind Bax favoring their anchoring to the mitochondrial membrane, a process that is required for the activation of MAP-1 and the release of cytochrome *c* (44–46). Bid activation was analyzed by Western blotting using a rabbit anti-Bid antibody that detects both the cleaved (active) and native forms of Bid (31). Active Bax was detected by immunoprecipitation with the anti-Bax antibody (clone 6A7), which reacts only with Bax in its conformationally active state, followed by Western blotting with the same antibody as described previously (27, 32). Other forms of Bax were detected by Western blotting of the samples with the anti-Bax antibody (clone 6A7). Our results indicate that neither H_2_O_2_ nor thapsigargin induce Bax activation; however, association of Bax and Bid is induced by both H_2_O_2_ and thapsigargin, as well as Bid activation under the same treatments in HL-60 cells. The effects of the treatments in HL-60 cells are in agreement with Lee (47) who probed that H_2_O_2_ is not able to alter Bax activity, or even, other anticancer drugs that have been evaluated in this cell model, such as furanodiene, which is only able to alter Bid activity, meanwhile other Bcl-2 family members like Bax or Bcl-2 remain unaltered (48). However, other results suggest that Bax is activated and translocated to the mitochondria by H_2_O_2_ in SW480 human colon adenocarcinoma cell line (49). This apparent discrepancy could be attributed to the different methodological procedures to determine Bax activation. In the present study we stimulated the cells with 100 µM for 120 minutes, while results shows by Nie *et al*. (49) were obtained after 6 hours of incubation, whereas under short time (6 hours) the endogenous Bax proapoptotic activity was not detected (49). All together, our results suggest that not only proapoptotic proteins Bax and Bid are less active in resting HL-60 cells than in cells where calcium reuptake has been inhibited, or in cells treated with ROS, but also that cell treatment with both agents induced activation of the transduction and expression mechanism of both proapoptotic proteins, which eventually resulted in a cellular death by apoptosis.

## CONCLUSIONS

The data presented here show the oxygen radical H_2_O_2_ and the inhibitor of calcium reuptake thapsigargin cause activation of caspase-3 and -9. Our studies also demonstrate that apoptotic events induced by both treatments are mainly of mitochondrial origin and are able to increase cell death by apoptosis and necrosis. We conclude that intracellular calcium depletion by treatment with H_2_O_2_ or thapsigargin, may induce the mitochondrial calcium-overload which in turn would generate more ROS which activate apoptotic phenomenon in the human myeloid cell line HL-60.
